# Comparative Mitogenomic Analysis and the Evolution of *Rhizoctonia solani* Anastomosis Groups

**DOI:** 10.3389/fmicb.2021.707281

**Published:** 2021-09-20

**Authors:** Runmao Lin, Yuan Xia, Yao Liu, Danhua Zhang, Xing Xiang, Xianyu Niu, Linjia Jiang, Xiaolin Wang, Aiping Zheng

**Affiliations:** ^1^Institute of Vegetables and Flowers, Chinese Academy of Agricultural Sciences, Beijing, China; ^2^Agriculture College, Sichuan Agricultural University, Chengdu, China; ^3^Rice Research Institute, Sichuan Agricultural University, Chengdu, China; ^4^State Key Laboratory of Crop Gene Exploration and Utilization in Southwest China, Chengdu, China

**Keywords:** *Rhizoctonia solani*, mitogenome, expression pattern, evolution, positive selection

## Abstract

Mitochondria are the major energy source for cell functions. However, for the plant fungal pathogens, mitogenome variations and their roles during the host infection processes remain largely unknown. *Rhizoctonia solani*, an important soil-borne pathogen, forms different anastomosis groups (AGs) and adapts to a broad range of hosts in nature. Here, we reported three complete mitogenomes of AG1-IA RSIA1, AG1-IB RSIB1, and AG1-IC, and performed a comparative analysis with nine published *Rhizoctonia* mitogenomes (AG1-IA XN, AG1-IB 7/3/14, AG3, AG4, and five *Rhizoctonia* sp. mitogenomes). These mitogenomes encoded 15 typical proteins (*cox1-3*, *cob*, *atp6*, *atp8-9*, *nad1-6*, *nad4L*, and *rps3*) and several LAGLIDADG/GIY-YIG endonucleases with sizes ranging from 109,017 bp (*Rhizoctonia* sp. SM) to 235,849 bp (AG3). We found that their large sizes were mainly contributed by repeat sequences and genes encoding endonucleases. We identified the complete sequence of the *rps3* gene in 10 *Rhizoctonia* mitogenomes, which contained 14 positively selected sites. Moreover, we inferred a robust maximum-likelihood phylogeny of 32 Basidiomycota mitogenomes, representing that seven *R. solani* and other five *Rhizoctonia* sp. lineages formed two parallel branches in Agaricomycotina. The comparative analysis showed that mitogenomes of Basidiomycota pathogens had high GC content and mitogenomes of *R. solani* had high repeat content. Compared to other strains, the AG1-IC strain had low substitution rates, which may affect its mitochondrial phylogenetic placement in the *R. solani* clade. Additionally, with the published RNA-seq data, we investigated gene expression patterns from different AGs during host infection stages. The expressed genes from AG1-IA (host: rice) and AG3 (host: potato) mainly formed four groups by k-mean partitioning analysis. However, conserved genes represented varied expression patterns, and only the patterns of *rps3-nad2* and *nad1-m3g18*/*mag28* (an LAGLIDADG endonuclease) were conserved in AG1-IA and AG3 as shown by the correlation coefficient analysis, suggesting regulation of gene repertoires adapting to infect varied hosts. The results of variations in mitogenome characteristics and the gene substitution rates and expression patterns may provide insights into the evolution of *R. solani* mitogenomes.

## Introduction

The basidiomycetous fungus *Rhizoctonia solani* Kühn [teleomorph *Thanatephorus cucumeris* (Frank) Donk] is a worldwide prevalent soil-borne plant pathogen. It causes diseases in many economically important crops (including rice, corn, soybeans, potatoes, wheat, cabbage, lettuce, sugar beets, and tomatoes), ornamental plants, and forest trees (Ogoshi, 1987; Gonzalez Garcia et al., 2006; Yang and Li, 2012; Molla et al., 2020).

The multinucleate *R. solani* isolates are grouped in the taxa within the *Rhizoctonia* species complex (Carling, 1996). These *R. solani* isolates are classified into 14 distinct anastomosis groups (i.e., AG1-AG13 and AGBI), and AG1 consists of four primary intraspecific subgroups of AG1-IA, AG1-IB, AG1-IC, and AG1-ID (Pannecoucque and Höfte, 2009; Yang and Li, 2012). Among AG1-AG13, strains are generally capable of fusing hyphae only in each AG, while strains from AGBI are capable of fusing hyphae with strains from 14 AGs (Sneh et al., 1991; Gonzalez Garcia et al., 2006). Meanwhile, the binucleate *Rhizoctonia* sp. isolates are classified in other taxa within the complex (Carling, 1996), which include 21 AGs (AG A-U).

In recent years, *R. solani* pathogenesis has been studied at the genomic and transcriptomic level, including AG1-IA that causes sheath blight in rice (*Oryza sativa*), corn (*Zea mays*), and soybeans (*Gl*ycine max; Zheng et al., 2013; Nadarajah et al., 2017; Xia et al., 2017; Yamamoto et al., 2019; Lee et al., 2021; Li et al., 2021a), AG1-IB that infects lettuce (Wibberg et al., 2015; Verwaaijen et al., 2017), AG3 that infects potatoes (Cubeta et al., 2014; Patil et al., 2018; Zrenner et al., 2020), and AG8 that infects wheat (Hane et al., 2014).

Mitochondrial genomes evolve independently of the nuclear genomes, and comparative mitogenome analysis sheds light on mitochondrial evolution (Gray, 2012). The relatively small size and mostly uniparental inheritance of fungal mitochondria also makes them ideal candidates for evolution, fungicide insensitivity, population genetics, and taxonomy studies (Bullerwell and Lang, 2005). So far, more than 800 complete fungal mitogenomes are available in the NCBI database^1^, providing a rich picture of their prevailing features, ancestral characteristics, and evolutionary trends. About 16% of these mitogenomes are in Basidiomycetes, including AG1-IB 7/3/14 and AG3 mitogenomes (Wibberg et al., 2013; Losada et al., 2014). The partial mitogenomes of AG1-IA and AG8 have also been reported (Zheng et al., 2013; Hane et al., 2014). The AG3 mitogenome revealed the expansion of mobile elements in *R. solani* and the synteny among AG1-IA, AG1-IB, and AG3 mitogenomes (Losada et al., 2014). Recently, multi-, bi-, and uninucleate *Rhizoctonia* mitogenomes have been reported (Li et al., 2021b). However, lack of complete mitogenomes of AG1-IA and AG1-IC that can infect cabbage and soybeans (Fu et al., 2014; Misawa and Aoki, 2017) prevents our understanding of the diversity of mitogenomic characteristics in *R. solani*. A comparison of mitogenomes in Basidiomycota could provide valuable insight into the origin and evolution of their complex mitogenomic features.

In fungal biology, mitochondria play a significant role in fungal virulence and adaptation (Ingavale et al., 2008; Chatre and Ricchetti, 2014; Calderone et al., 2015; Sun et al., 2019). Previous studies show that mutations in the mitogenome of the tree pathogen *Cryphonectria parasitica* weaken its virulence (Monteiro-Vitorello et al., 1995), and the mitochondrial cytochrome C from the animal pathogen *Aspergillus fumigatus* is critical for its virulence (Grahl et al., 2012). For the human opportunistic pathogen *Cryptococcus neoformans*, the changes in its mitochondria morphology by fission and fusion could dramatically influence its virulence (Chang and Doering, 2018). Meanwhile, the mitochondria of *C. neoformans* play a key role in hypoxia adaptation (Ingavale et al., 2008). Moreover, lineage-specific adaptations in mitochondria have been found to be associated with hosts in another opportunistic pathogen, *Candida albicans*, and the mitochondrial proteins influence *C. albicans* respiration (Sun et al., 2019) that is required for its growth, morphogenesis, and virulence. Many chemicals can efficiently inhibit respiration in *C. albicans* while not damaging the mammalian host (Duvenage et al., 2019), which may be a strategy to develop a target for antifungals in the future studies.

The fungal mitogenomes may be a powerful system to measure adaptation at the molecular level. The estimation of substitution rates may provide evidence of adaptive evolution that possibly affects only a few amino acids at a few time points (Yang and Nielsen, 2002). To measure the selection pressure on amino acid replacement mutation of protein-coding genes, the method of calculating the non-synonymous/synonymous substitution rate ratio (dN/dS) is widely used (Nielsen and Yang, 2003). Based on eight mitogenomes of the *Synalpheus* species of non-eusocial and eusocial groups, the comparative analyses of synonymous substitution rates and selection signals provide direct evidence of eusociality impacting genome evolution (Chak et al., 2021). The discovery of several positively selected sites in eusocial lineages may represent adaptation (Chak et al., 2021). For host specificity of *R. solani* AG strains, the examination of substitution rates in mitogenomes may help to reveal their adaptability to hosts.

Additionally, the expression of *R. solani* nuclear genes during host infection enhances our discovery of pathogenic factors, including candidate effectors (Zheng et al., 2013). The interactions between AG1-IA and rice, AG1-IB and lettuce, as well as AG3 and potatoes have been explored (Zheng et al., 2013; Verwaaijen et al., 2017; Xia et al., 2017; Zrenner et al., 2020), providing an avenue to investigate the expression of mitochondrial encoded genes and their roles during infection process. A schematic of varied gene expression patterns during infecting different hosts may provide clues to understand fungal adaptation to hosts.

Here, in exploring the evolution and host adaptation in *R. solani* by performing comparative analysis of mitogenomes, we reported three complete mitogenomes of *R. solani* AG1-IA RSIA1, AG1-IB RSIB1, and AG1-IC, providing a resource for revealing mitogenome characteristics. We also investigated the phylogenetic analysis and selection pressure analysis on amino acids, which may indicate significant sites contributing to adaptation, and examined the varied expression patterns of encoded genes from mitogenomes of AG strains with host specificity during infection, which may further provide knowledge about host adaptation in *R. solani*.

## Materials and Methods

### Sampling and DNA Extraction

The *R. solani* AG1-IA RSIA1, AG1-IB RSIB1, and AG1-IC strains were provided by Prof. Erxun Zhou at South China Agricultural University. The strains were grown in potato dextrose broth medium at 28°C, and the genomic DNA was extracted using a modified CTAB method (Ciampi et al., 2008). All the *Rhizoctonia* mitogenomes used in this study were listed in Supplementary Table 1, including the previously reported mitogenomes of AG1-IB 7/3/14 (Wibberg et al., 2013), AG3 (Losada et al., 2014), AG4 (Zhang et al., 2021), AG1-IA XN, *R. cerealis* RW, and four *Rhizoctonia* sp. strains, JN, LY, SM, and YR (Li et al., 2021b). The hosts of five complete mitogenomes were listed in Table 1.

**TABLE 1:** Statistics of mitochondrial genomes of *Rhizoctonia solani* species.

	AG1-IA RSIA1	AG1-IB RSIB1	AG1-IB 7/3/14	AG1-IC	AG3-PT
Complete genome	Yes	Yes	Yes	Yes	Yes
Genome size (bp)	152,549	168,442	162,751	175,227	235,859
Typical proteins^a^	15	15	15	15	15
Other proteins^b^	36	12	15	24	21
tRNA	26	26	25	26	27
rRNA	2	2	2	2	2
GC content (%)	33.7	36.5	36.4	34.5	35.9
Repeat sequences (%)	17.86	32.17	30.52	28.96	42.73
Accession	MW995474.1	MW995475.1	HF546977.1	MW995476.1	KC352446.1
Reference	This study	This study	Wibberg et al., 2013	This study	Losada et al., 2014
Host^c^	Rice, corn, soybeans, barley, sorghum, potatoes, peanut, cabbage, leaf lettuce, et al.	Corn, sugar beets, gay feather, common bean, soybeans, cabbage, leaf lettuce et al.		Sugar beets, carrot, buckwheat, flax, soybeans, bean, cabbage, pineapple, panicum, spinach and radish	Potatoes with black scurf symptoms

*^a^The 15 genes include cytochrome c oxidase subunits (cox1, cox2, and cox3), apocytochrome b (cob), ATP synthase subunits (atp6, atp8, and atp9), subunits of NADH dehydrogenase (nad1, nad2, nad3, nad4, nad4L, nad5, and nad6), and one ribosomal protein (rps3).*

*^b^LAGLIDADG homing endonucleases and GIY-YIG endonucleases.*

*^c^The host information was inferred from study by Yang and Li (2012).*

### Mitogenome Assembly and Annotation

For the sequenced PacBio RS long reads of AG1-IA RSIA1, AG1-IB RSIB1, and AG1-IC strains, we used LoRDEC v0.5 (Salmela and Rivals, 2014) with parameters of “-k 19 -s 3” for read correction based on Illumina short reads with insert size of ∼180 bp. Then we used Canu v1.2 (Koren et al., 2017) with default parameters for genome assembly, which generated the complete three mitogenomes of AG1-IA, AG1-IB, and AG1-IC. We examined the circular map of the mitogenomes and improved the sequences using Pilon v1.17 (Walker et al., 2014) with default parameters.

From the mitogenome sequences, we predicted and annotated the 15 typical protein-coding genes (seven subunits of NADH dehydrogenase, three cytochrome c oxidase subunits, three ATP synthase subunits, one apocytochrome b, and one ribosomal protein) and other protein-coding genes (LAGLIDADG homing endonucleases and GIY-YIG endonucleases) by the pipeline as follows. First, we aligned the mitogenome sequences against amino acids in the NCBI NR database using BLASTPX with an *E*-value cutoff of 1e-10, which detected candidate reference genes from the NR database. Then, we used Exonerate v2.2.0 (Slater and Birney, 2005) with the “protein2genome” model to predict genes by aligning mitogenome sequences against these candidate reference genes. We found that some Exonerate-predicted genes may be incomplete without considering the start and/or stop codons. For each predicted gene, we wrote an in-house Perl script to check and improve the prediction by scanning its up-/down-stream genomic sequences to identify the start and stop codons. For each gene region, Exonerate may predict multiple candidate genes because of multiple NR reference genes being used for alignment analysis. All candidate genes were aligned to NR reference genes again using BLASTP, which could be useful for manual examination of the length and *E*-value for each predicted gene. For multiple predicted genes from the same genomic region, we manually selected the one with the low BLASTP *E*-value and with similar length compared to the NR reference genes. Finally, the annotation of selected genes was inferred from NR reference genes. For tRNA genes, we used tRNAscan-SE v1.3.1 (Lowe and Eddy, 1997) with translation table 4 for gene discovery and removed candidate tRNAs with types of “Undet” (i.e., without anticodons). The reported rRNA sequences in the SILVA database (Quast et al., 2013) were used as reference genes for rRNA annotation by performing BLASTN analysis.

We used the same method to annotate the encoding genes of the previously reported AG1-IB 7/3/14, AG3, AG4, AG1-IA XN, *R. cerealis* RW, and four *Rhizoctonia* sp. mitogenome sequences (JN, LY, SM, and YR). The AG4 mitogenome was included in the reported sequence deposited in NCBI with the accession number of JADHEA010000014.1 (Zhang et al., 2021; Supplementary Table 2). We found that there was one base deletion in the *rps3* gene (Supplementary Figure 1), preventing the prediction of the *rps3* gene in AG4 (Supplementary Table 3). As we could not be sure that the AG4 *rps3* was a real pseudogene or had an error in assembly sequence, we did not include AG4 *rps3* for comparative analysis. For the other six mitogenomes, we found that there were 17, 4, 1, 1, and 1 gap regions (i.e., “Ns” in assemblies) in XN (accession number: MT887631.1), LY (accession number: MT887629.1), SM (accession number: MT887628.1), YR (accession number: MT887627.1), and RW (accession number: MT887630.1) mitogenomes, respectively. The *Rhizoctonia* sp. JN (accession number: MT887626.1) mitogenome did not have a gap sequence, but its length (∼126 kb) was ∼35 kb less than that of *Rhizoctonia* sp. LY (∼161 kb), preventing the confirmation of complete mitogenome of *Rhizoctonia* sp. JN. The incomplete mitogenomes may prevent the prediction of genes (such as the incomplete *rps3* in RW strain). However, the complete sequences of 14 typical proteins (*cox1-3*, *cob*, *atp6*, *atp8-9*, *nad1-6*, and *nad4L*) were identified in 12 *Rhizoctonia* mitogenomes. To perform comparative analysis of endonucleases, only five complete mitogenomes of AG1-IA RSIA1, AG1-IB RSIB1, AG1-IC, AG1-IB 7/3/14, and AG3 were used (Table 1 and Supplementary Table 4).

We used the *de novo* method to identify repeat sequences in *Rhizoctonia* mitogenomes. The repeat library was constructed based on the mitochondrial genome sequences using RepeatScout v1.0.5 (Price et al., 2005). This library was used to identify repeat sequences using RepeatMasker v4.0.5^2^.

### Comparative Mitogenomic Analysis

Based on amino acid sequences of genes from five complete mitogenomes of *R. solani* strains, we used OrthoFinder 0.7.1 (Emms and Kelly, 2015) to detect their orthologous genes. The sequence alignment of the *rps3* gene was done by MUSCLE v3.8.31 (Edgar, 2004). The positively selected signals in *rps3* genes were detected using CODEML implemented in PAML v 4.8a (Yang, 2007), as described in the previous study (Lin et al., 2015). For the *rps3* gene with positively selected signals, we used PSIPRED (Buchan and Jones, 2019) and RoseTTAFold (Baek et al., 2021) to predict its protein structure.

The KaKs_Calculator 1.2 estimated dN and dS values using model-selected and model-averaged methods based on a group of models (Zhang et al., 2006). As in the description in the KaKs_Calculator study (Zhang et al., 2006), different substitution models considered different evolutionary features, resulting in different estimates, and for protein-coding sequences, the use of many features may lead to more reliable evolutionary information. We used the 10 methods (NG, LWL, MLWL, LPB, MLPB, GY-HKY, YN, MYN, MS, and MA) implemented in the KaKs_Calculator to estimate dN, dS, and dN/dS values for protein-coding genes in *Rhizoctonia* mitogenomes. We used their mean values to represent the increasing or decreasing trends of the dN, dS, and dN/dS values for the comparative analysis.

To perform phylogeny analysis for 32 mitogenomes from Basidiomycetes (Supplementary Table 1), we selected amino acids from 14 typical protein-coding genes (*cox1*, *cox2*, *cox3*, *cob*, *atp6*, *atp8*, *atp9*, *nad1*, *nad2*, *nad3*, *nad4*, *nad4L*, *nad5*, and *nad6*) and performed MUSCLE alignment. Then these sequences were concatenated for the following analysis. The ProtTest v3.4 (Darriba et al., 2011) with parameters of “-all-distributions -F -AIC -BIC” identified the best model of LG + I + G + F for constructing the maximum-likelihood phylogeny. Then we used Mega v6.06 (Tamura et al., 2013) to build the maximum-likelihood phylogenetic tree with bootstrap value of 1,000.

### Transcriptomic Analysis

For AG1-IA, its gene expression analysis was investigated using RNA-seq after rice infection at 10 h (10-h), 18, 24, 32, 48, and 72-h (Zheng et al., 2013). The RNA-seq data before and after infecting different crops (i.e., rice, corn, and soybeans) of different AG1-IA strains that were isolated from rice, corn, and soybeans have been reported (Xia et al., 2017). For three strains of AG3, their interaction with potato sprouts after infection of three and 8 days were investigated by transcriptomic analysis (Zrenner et al., 2020). For the reported RNA-seq data, we analyzed data from each study independently. We calculated the gene expression FPKM (fragments per kilo base per million mapped reads) values following the protocol (Pertea et al., 2016) using HISAT2 (Kim et al., 2019), StringTie (Pertea et al., 2015), and Ballgown (Frazee et al., 2015) software. Based on the expression values, we used the R function of fviz_cluster that was implemented in the factoextra package to detect gene clusters and used the R function cor to calculate the Pearson correlation coefficient between genes.

### Gene Expression Analysis via Real-Time Quantitative Reverse Transcription PCR (RT-qPCR)

A total of 15 genes were selected for RT-qPCR analysis. First-strand cDNA was synthesized from total RNA using HiScript II Q RT Supermix for qPCR with a gDNA wiper (Vazyme R223-01, Nanjing, China). RT-qPCR was performed using the AceQ qPCR SYBR green master mix (Vazyme Q111-02/03, Nanjing, China). The qPCR reactions were performed in a final volume of 10 μL containing 5 μL of 2 × AceQ qPCR SYBR green master mix, 0.25 μL of 10 μM of each primer, 4.25 μL of ddH_2_0, and 0.25 μL of cDNA. Reactions were carried out at 95°C for 5 min, followed by 40 cycles of 95°C for 10 s, 60°C for 30 s, and melting curve analysis from 60°C to 95°C at 1°C increments (qTOWER^3^G, Jena German). Primers for qPCR were designed based on our predicted gene sequences by NCBI primer blast, and the parameters were modified to suit the RT-qPCR conditions (Supplementary Table 5). The 18S gene was used as an internal control. Fold changes were determined by the 2^–Δ^ ^Δ^ ^Ct^ method. All qPCR reactions were run in triplicate.

## Results

### General Characteristics of *R. solani* Mitogenomes

**FIGURE 1:**
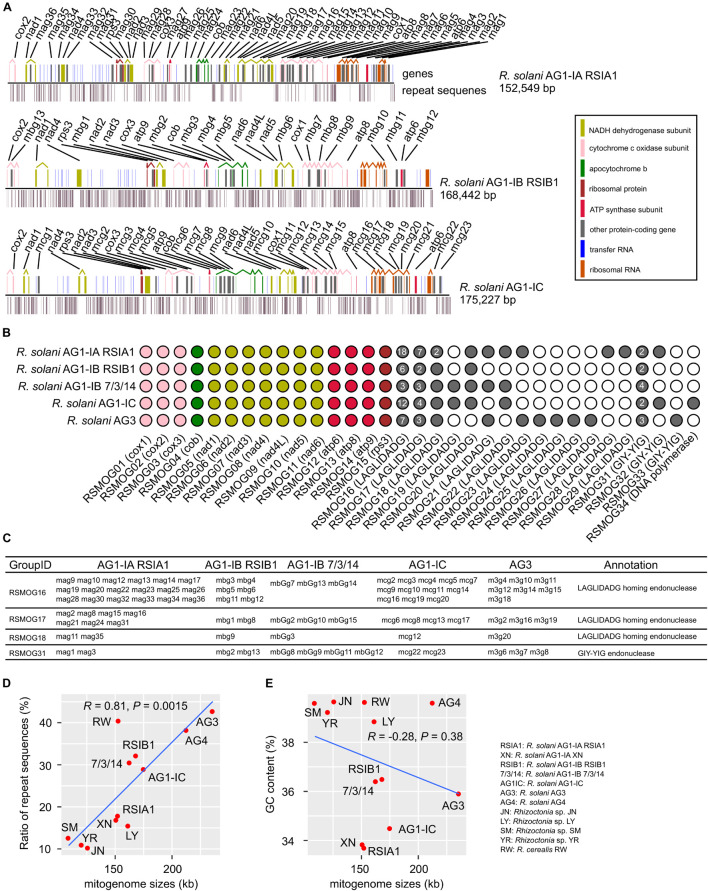
Characterization of mitogenomes in *Rhizoctonia solani*. **(A)** Maps of six mitogenomes. The genomic positions of genes and repeat sequences were shown. Many protein-coding genes contained multiple exons that were connected by broken lines. **(B)** Orthologous groups of protein-coding genes in mitogenomes. **(C)** Four groups contained multiple genes. **(D,E)** Distribution of repeat sequences and GC contents in *Rhizoctonia* mitogenomes. In the MATERIALS AND METHODS section (Mitogenome assembly and annotation), we described selecting *Rhizoctonia* mitogenomes for comparative analysis. The R functions (ggplot and stat_cor with the Pearson correlation coefficient method implemented in ggplot2 and ggpubr packages, respectively) were used to draw the **(D,E)**.

Here we reported three complete mitogenomes of *R. solani* AG1-IA, AG1-IB, and AG1-IC, with the sizes of ∼152-168 kb (Table 1), and performed a comparative analysis with two published complete mitogenomes of *R. solani* AG1-IB and AG3 (Wibberg et al., 2013; Losada et al., 2014). Among the five complete mitogenomes, the smallest size was ∼152 kb in AG1-IA and the largest size was ∼235 kb in AG3 (Table 1). We found highly conserved sequences in the two AG1-IB mitogenomes of RSIB1 and 7/3/14 strains. These mitogenomes consisted of an essential set of 15 typical protein-coding genes (three cytochrome c oxidase subunits: *cox1*, *cox2*, *cox3*; the apocytochrome b: *cob*; three ATP synthase subunits: *atp6*, *atp8*, *atp9*; seven subunits of NADH dehydrogenase: *nad1*, *nad2*, *nad3*, *nad4*, *nad4L*, *nad5*, *nad6*; and one ribosomal protein: *rps3*), LAGLIDADG homing endonucleases and GIY-YIG endonucleases (ranging from 12 in AG1-IB and 36 in AG1-IA), and the small and large ribosomal RNA subunits (*rns*, *rnl*), and tRNAs (Table 1, Figure 1, and Supplementary Table 4). All protein-coding genes were clustered into 15, 14, 3, and 1 orthologous groups for 15 typical protein-coding genes, LAGLIDADG homing endonucleases, GIY-YIG endonucleases and DNA polymerase, respectively (Figure 1B). Most groups contained single-copy genes from each mitogenome, excluding three LAGLIDADG groups and one GIY-YIG group that each contained multiple-copy genes (Figure 1C). For example, the RSMOG01 group contained only one *cox1* in each strain, while the RSMOG16 group contained LAGLIDADG homing endonucleases ranging from 3 in AG1-IB 7/3/14 to 18 in AG1-IA. Compared to other strains, AG1-IA contained more LAGLIDADG homing endonucleases that were mainly encoded in the intron regions of *rnl*, *cox1*, and *nad4L* (Figure 1A and Supplementary Table 4).

Among these mitogenomes, there were 27,239 (17.86%), 54,190 (32.17%), 49,669 (30.52%), 50,748 (28.96%), and 100,785 (42.73%) bp of repeat sequences in AG1-IA, AG1-IB, AG1-IB 7/3/14, AG-IC, and AG3, respectively (Supplementary Tables 6–10), with the lowest and highest ratios in AG1-IA and AG3, respectively. The genomic size of AG3 was 83,310 bp larger than that of AG1-IA (Table 1), while the repeat sequences of AG3 were 73,546 bp larger than those of AG1-IA, indicating that repetitive sequences contribute to the large size of the AG3 mitogenome. The comparison of genomic sizes and ratios of repeat sequences suggested their positive correlations (*R* = 0.81, *P* = 0.0015), i.e., longer genomic sizes containing more repetitive sequences (Figure 1D).

However, the relationship between GC contents and genomic sizes was not similar to that between repeat sequences and genomic sizes (Figure 1E). Although AG3 had the largest genomic size, its GC content was larger than 33.7% of the GC content in AG1-IA and was lower than 36.5% for AG-IB (Table 1). The distribution of GC content among mitogenomes may suggest the different sequence preferences in mitogenomes.

### Mitochondrial Phylogenetic Relationships Between *R. solani* and Other Fungi in Basidiomycota

**FIGURE 2:**
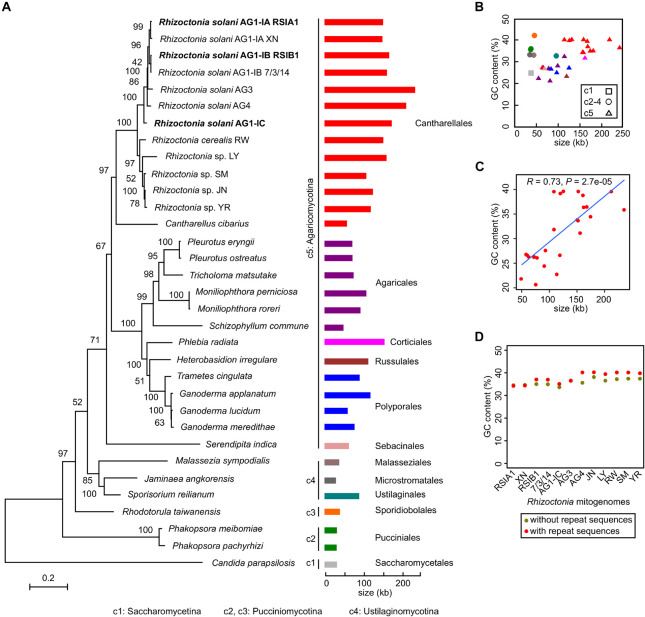
Phylogenetic relationships between *Rhizoctonia solani* and other fungi in Basidiomycota. **(A)** The mitogenome phylogeny of 33 fungal strains. The *Candida parapsilosis* in Ascomycete was used as the outgroup. The maximum-likelihood bootstrap values were shown. The sizes of mitogenomes were shown with different colors for strains from different orders. The newly reported complete mitogenomes were marked in bold. **(B)** Distribution between sizes and GC content from 33 fungal mitogenomes. **(C)** Positive relationship between GC content and mitogenomic sizes for Agaricomycotina mitogenomes. **(D)** Distribution of GC content in *Rhizoctonia* mitogenomes with and without repeat sequences.

Based on the complete mitogenome of *R. solani*, we explored their phylogenetic relationships with other fungi. A phylogeny for 32 fungal strains in Basidiomycota and one strain in Ascomycota as an outgroup was constructed, which represented 26, 2, and 3 Basidiomycetes strains in three subphyla of Agaricomycotina, Pucciniomycotina, and Ustilaginomycotina, respectively (Figure 2A). *Rhizoctonia* strains were in Agaricomycotina. The seven multinucleate *R. solani* strains (AG1-IA RSIA1 and XN, AG1-IB RSIB1 and 7/3/14, AG1-IC, AG3, and AG4) were parallel with a clade containing two binucleate *Rhizoctonia* strains (*Rhizoctonia* sp. LY and *R. cerealis* RW) and three uninucleate *Rhizoctonia* strains (*Rhizoctonia* sp. SM, JN, and YR; Li et al., 2021b). These *Rhizoctonia* lineages were in the Cantharellales order, plus the *Cantharellus cibarius* lineage formed one large clade that was parallel with another clade containing 12 lineages in four orders (Agaricales, Corticiales, Russulales, and Polyporales). Outside the branches of these 25 lineages, there was one branch for *Serendipita indica* in the Sebacinales order (in Agaricomycotina).

From the phylogeny, mitochondrial genomic sizes varied from 29 kb (*Jaminaea angkorensis* strain; in Microstromatales, Ustilaginomycotina) to 235 kb (AG3; in Cantharellales, Agaricomycotina). We found that five strains from Pucciniomycotina and Ustilaginomycotina had mitochondrial genomic sizes of less than 41 kb (Supplementary Table 1), excepting the *Sporisorium reilianum* strain of ∼90 kb in size (in Ustilaginales, Ustilaginomycotina). However, the mitochondrial genomic sizes were obviously increased in strains from Agaricomycotina, a separate clade in Basidiomycota, especially for *R. solani* strains in Cantharellales and *Phlebia radiata* strain in Corticiales, with sizes larger than 150 kb (Figure 2 and Supplementary Table 1).

Considering both GC content and mitogenomic sizes, we found that in Pucciniomycotina and Ustilaginomycotina, most mitogenomes had small sizes but had high GC content (>31%). In Agaricomycotina, the GC content was quite different, ranging from 22.8 to 39.66% (Figures 2B,C and Supplementary Table 1). A positive relationship (*R* = 0.73, *P* = 2.7e-05) between GC content and mitogenomic sizes are shown for Agaricomycotina strains, i.e., strains with higher genomic sizes with higher GC content (Figure 2C). Meanwhile, the repeat sequences in the mitogenomes had little effect on GC content (Figure 2D). Moreover, in Basidiomycota fungi, high mitochondrial GC content was found in pathogens (including *Phakopsora* sp. in Pucciniomycotina, *Malassezia* sp., and *Sporisorium* sp. in Ustilaginomycotina, and *Rhizoctonia* sp. in Agaricomycotina; Figure 2 and Supplementary Table 1).

### Changes in Non-Synonymous and Synonymous Substitution Rates (dN and dS) of *R. solani* Mitogenomes

**FIGURE 3:**
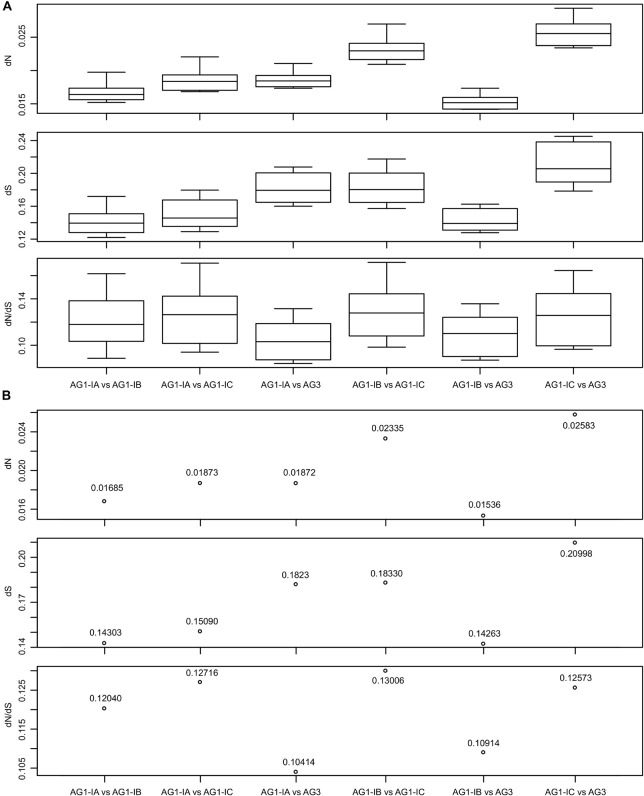
The dN, dS, and dN/dS values for each pair of *Rhizoctonia solani* mitogenomes. **(A)** The box plots displayed the values estimated by 10 methods implemented in the KaKs_Calculator. The analysis was done based on concatenated sequences of 15 typical protein-coding genes. **(B)** The mean of the estimated values shown in **(A)**.

Although the *R. solani* phylogeny formed one branch in the mitochondrial phylogeny (Figure 2A), the AG1-IC and other AG1 strains were separated by the AG3 strain, which may reflect the sequence changes in mitogenomes. We used the KaKs_Calculator to calculate the dN and dS values for the concatenated sequences of 15 typical protein-coding genes and found that all dN, dS, and dN/dS values were lower than 0.03000, 0.25000, and 0.18000, respectively (Figure 3 and Supplementary Table 11). For each pair of mitogenomes, the AG1-IC and AG3 mitogenomes had the highest dN values (i.e., 0.02339∼0.02934, mean 0.02583), while AG1-IB and AG3 had the lowest dN values (i.e., 0.01418∼0.01735, mean 0.01536). Similar results were found for dS values, i.e., the highest dS values were found for AG1-IC vs AG3 and the lowest dS values were found for AG1-IB vs AG3. The mitogenome pairs of AG1-IC and other *R. solani* mitogenomes showed the higher substitution rates than those from mitogenome pairs without AG1-IC (Figure 3 and Supplementary Table 11), supporting the phylogenetic topology for *R. solani* mitogenomes (Figure 2A).

### Discovery of Positively Selected Sites in *Rhizoctonia rps3* Genes

**FIGURE 4:**
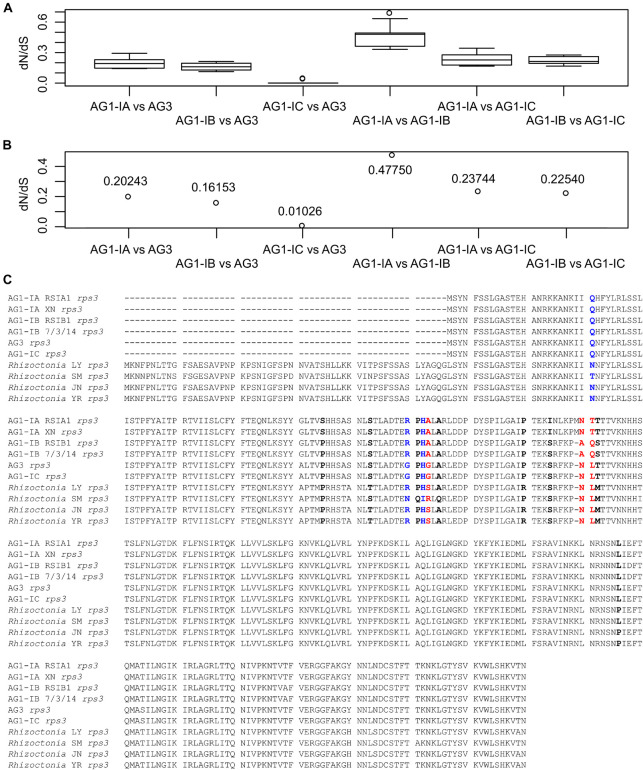
Detection of positively selected sites in *rps3*. **(A)** The dN/dS values for each pair of *rps3* genes from mitogenomes. The dN/dS values were calculated by 10 methods implemented in the KaKs_Calculator. **(B)** The mean of the estimated values shown in **(A)**. **(C)** Display of positively selected sites in *rps3* genes. The 14 positively selected sites were detected by the CODEML program in PAML, including three sites (in blue) with *P* > 95% and three sites (in red) with *P* > 99%.

For each of 15 typical genes, we calculated their dN/dS values and found that all of them were less than one, including the *rps3* genes (Figures 4A,B). The amino acid (aa) sequences of *rps3* in four strains (AG1-IB RSIB1 and 3/7/14, AG1-IC, and AG3) were 283 aa, and one more aa was found in AG1-IA strains (RSIA1 and XN), as well as 56 more aa were found in *Rhizoctonia* sp. strains (LY, SM, JN, and YR). The sequence alignment showed that they shared sequence identities larger than 89%, suggesting the conserved sequences in *Rhizoctonia rps3* genes. However, with the CODEML method in PAML (Yang, 2007), we detected 14 positively selected sites (Figure 4C). Among them, five sites (“RPHA” and “A” in AG1-IA) were closely linked with each other (aa position: 84–89), with one amino acid (“L” in AG1-IA) flanking these positively selected sites; meanwhile, for the 27 aa downstream of these sites, other positively selected sites were found, including “P,” “I,” and “NTT” in AG1-IA (Figure 4C). These changed sites represented five types of different sequences in AG1-IA, AG1-IB, AG1-IC/AG3, SM, and other strains (LY, JN, and YR), respectively, which were related to their phylogenic topology (Figure 2A). The secondary structure of the *rps3* gene showed that the contiguous sites with positively selected signals were in the helix and coil regions (Supplementary Figure 2A). For these sites, we marked them in the predicted protein structure (with RoseTTAFold confidence of 0.46) as well (Supplementary Figure 2B).

### Expression Patterns of Genes in *R. solani* Mitogenomes During Host Infection

**FIGURE 5:**
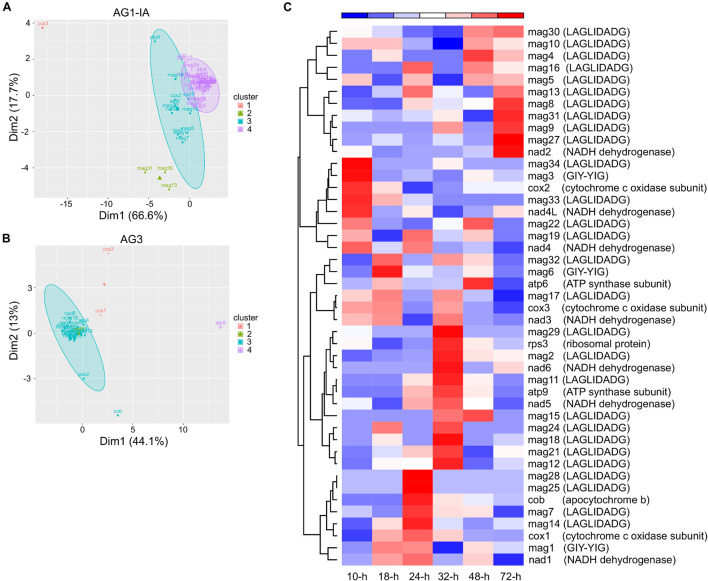
The expression patterns of genes from *Rhizoctonia solani* mitogenomes. **(A,B)** The distribution of gene clusters from AG1-IA and AG3. **(C)** The heatmap of AG1-IA genes after infecting rice at 10, 18, 24, 32, 48, and 72 h.

From RNA-seq for fungi-host interactions (i.e., interactions between AG1-IA and rice, soybeans, corn; AG1-IB and lettuce; AG3 and potatoes; Zheng et al., 2013; Xia et al., 2017; Zrenner et al., 2020), we analyzed the expression patterns of mitochondrial genes, which may suggest their roles during host infection. Based on gene expression FPKM values, 51 and 27 expressed genes from AG1-IA and AG3 mitogenomes, respectively, were all clustered into four clusters (Figures 5A,B and Supplementary Tables 12,13). Not all of each functional group of genes cytochrome c oxidase subunit, ATP synthase subunit, NADH dehydrogenase subunit, LAGLIDADG endonuclease, and GIY-YIG endonuclease were clustered into the same groups. For example, *cox1*, *cox2*, and *cox3* from AG1-IA were clustered into three groups. The gene clusters showed different expression patterns after infecting hosts.

We further explored gene expression during rice infection, which displayed varied gene expression peaks (Figure 5C). The peaks for *cox1*, *cox2*, and *cox3* from AG1-IA mitogenome were at 24 (i.e., 24 h), 10, and 18-h, respectively, although they all had functional cytochrome c oxidase subunits. Similarly, the peaks for *atp6* and *atp9* were at 48 and 32-h, respectively, and *atp8* was not expressed during rice infection. Meanwhile, LAGLIDADG endonucleases and GIY-YIG endonucleases represented expression peaks after host infection. For example, *mag2* (a LAGLIDADG endonuclease) displayed an expression pattern similar to that of *nad6*, with the peak at 32-h; mag6 (a GIY-YIG endonuclease) showed a peak at 18-h. Similarly, peaks for different genes from AG3 mitogenomes during potato infection were found as well (Supplementary Figure 3).

### Correlations Between Expressed Genes in *R. solani* Mitogenomes During Host Infection

**FIGURE 6:**
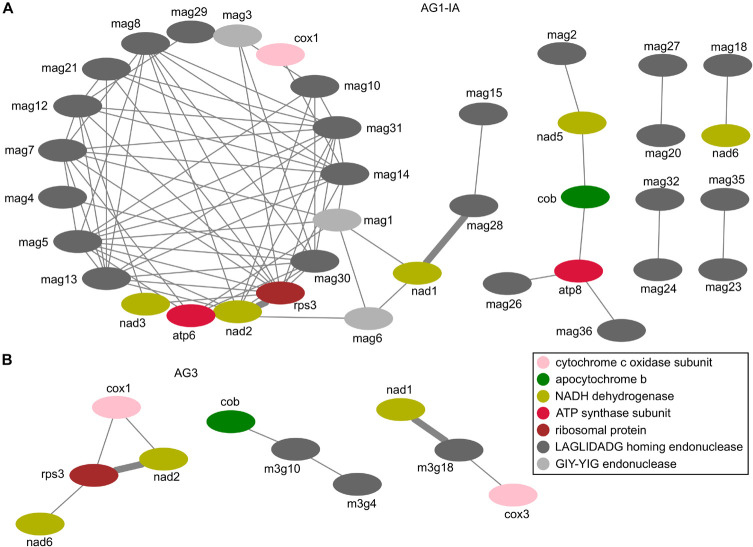
Correlation coefficients between each pair of genes encoded in mitogenomes. **(A)** The correlations for genes in AG1-IA. **(B)** The correlations for genes in AG3.

For the expressed genes (including 15 typical proteins, LAGLIDADG endonucleases, and GIY-YIG endonucleases), we calculated the Pearson correlation coefficient to measure their expression similarity. With the Pearson correlation coefficient value cutoff of 0.6, we identified 76 and 8 positive correlations between each pair of genes in AG1-IA and AG3, respectively (Supplementary Tables 14,15). Among these genes, *cox1*, *cob*, *nad1*, *nad2*, *nad6*, *rps3*, and LAGLIDADG endonucleases in RSMOG16 orthologous group (11 genes in AG1-IA: *mag10*, *mag12*, *mag13*, *mag14*, *mag20*, *mag23*, *mag26*, *mag28*, *mag30*, *mag32*, *mag36*; three genes in AG3: *m3g4*, *m3g10*, and *m3g18*; Supplementary Table 4) were found in both AG1-IA and AG3. To view their relationships clearly, we chose the network to display these correlated pair of genes. The different topologies for these correlations were shown in AG1-IA and AG3 (Figure 6), with only two conserved correlations (i.e., *nad2-rps3* and *nad1-mag28*/*m3g18*). In the neighboring genes *nad2* and *rps3* there were some repeat sequences; however, these repeat sequences from AG1-IA and AG3 mitogenomes were not similar sequences detected by BLAST alignment. Because the correlation coefficient was inferred from gene expression during varied host infection (i.e., rice infection for AG1-IA and potato infection for AG3; Supplementary Tables 12,13), there were different network topologies for expressed genes (Figure 6), which showed their different expression profiles in AG1-IA and AG3 mitogenomes, possibly indicating the different roles for these genes after AG1-IA and AG3 mitogenomes separated from the same ancestor.

## Discussion

It has been proposed that mitochondria evolved from free-living bacteria via symbiosis within a eukaryotic host cell (Margulis, 1970). With that in mind, we compared examples of GC content variations in the bacterial kingdom with those we have observed in some of our fungal mitogenomes. In bacteria, the high genomic GC content is proposed to be associated with high rates of DNA damage and environmental factors (Wu et al., 2012; Weissman et al., 2019), and it is suggested to be maintained in some species by mutation pressure (Hildebrand et al., 2010). Considering both GC content and mitogenomic sizes, the Agaricomycotina clade is of interest. A comparison of mitogenomes between *Rhizoctonia* and other non-pathogens in Agaricomycotina showed that *Rhizoctonia* had a higher GC content, and a comparison of mitogenomes between *Rhizoctonia* and other plant pathogens that were separated early in Basidiomycota showed that *Rhizoctonia* had more repeat sequences. The results may indicate the divergent evolution of Basidiomycota mitogenomes. The high GC content in mitogenomes of *Rhizoctonia* that have broader host ranges may have evolved under pathogenic environmental pressure.

The comparative analysis of five complete *R. solani* mitogenomes showed the natural existence of varied mitogenomic characteristics in sizes, endonuclease genes (both LAGLIDADG and GIY-YIG endonucleases), and repeat sequences. The repeat sequences and endonucleases are the major contributors to the size variations. At least 12∼36 endonucleases were encoded in these mitogenomes (Table 1), and there were 73,546 bp (31.18%) repeat sequences in the AG3 mitogenome, more than those in AG1-IA (Supplementary Tables 6,10). In fungal mitogenomes, multiple repeat sequences are the main cause of size expansion in mitogenomes (Losada et al., 2014; Li et al., 2015). The genes encoding endonucleases are considered mobile genetic elements that invaded introns and intergenic sequences, and they have been found to play an important role in causing mitogenome size variation (Kolesnikova et al., 2019).

LAGLIDADG and GIY-YIG endonucleases have been found in fungal mitogenomes belonging to orders in all fungal phyla (Belfort et al., 2002; Megarioti and Kouvelis, 2020). These endonucleases possess special conserved amino acid motifs and are encoded in the intron regions of fungal mitogenomes (Stoddard, 2014). The LAGLIDADG endonuclease has the ability to recognize 18–22 bp target sequences (Belfort and Roberts, 1997; Chevalier et al., 2005). These endonucleases may originate from free-standing open reading frames, and endonucleases and their intron hosts may have co-evolved through recombination and horizontal gene transfer (Megarioti and Kouvelis, 2020). Yeast endonucleases have been found to drive recombination of protein-coding genes (Wu and Hao, 2019). Currently, only five complete mitogenomes in *R. solani* have been reported. With the increasing release of genomic data, the evolution of endonucleases in *R. solani* will be explored in the future studies.

Meanwhile, the expression peaks of endonucleases during host infection were identified (Figure 5C), such as the high expression of *mag3* (a GIY-YIG endonuclease, located within the intergenic region between *atp6* and *rns*) and *mag33* (a LAGLIDADG endonuclease, located within the intergenic region between *nad4* and *rps3*) at 10-h after rice infection. The expression patterns of *mag31* (a LAGLIDADG endonuclease) and *rps3* containing the intron host of *mag31* were different, i.e., with expression peaks at 72 and 32-h, respectively, indicating that endonuclease and its inserted gene were expressed independently. These expression peaks may suggest the significant roles of endonucleases during host infection and independent roles for invasive endonucleases/introns and *rps3* genes. As fungal mitochondria acting as organelles to provide energy for cell functions, their encoded genes displayed varied expression peaks after infection, indicating significant cooperation among these genes.

The analysis of interspersed repeat sequences in the AG3 mitogenome suggested that the stable secondary structures exhibited by repeats may comprise catalytic RNA elements (Losada et al., 2014). None of the repeat sequences were shared between AG3 and AG1-IB 7/3/14 or between AG3 and other fungal mitogenomes in Basidiomycota, suggesting the unique evolutionary phenomenon of repeat acquisition in *R. solani* (Losada et al., 2014). The mitochondrial repeat sequences had been considered as putative elements for recombination or regulation (Ghikas et al., 2006). For both complete AG1-IB mitogenomes (AG1-IB RSIB1 and 7/3/14 in Figure 1A), their repeat sequence contents vary from each other, and these differences resulted in the size variation between AG1-IB and AG1-IB 7/3/14 mitogenomes (Supplementary Tables 7,8). Meanwhile, in the AG1-IA mitogenome, the repeat sequences may affect the expression of genes because similar repeat sequences nearby the each pair of genes (*rps3-nad2* and *nad1-mag28*) with positive correlations in expression were found. However, the influence of repeat sequence on gene expression in mitogenomes is required to further evaluate.

Non-synonymous and synonymous substitution rates (dN and dS) were different for each pair of *R. solani* mitogenomes (Figure 3), which may affect the phylogenetic placement of AG1-IC in the phylogeny (Figure 2A) because it was far from AG1-IB branches. The conflict between mitochondrial (Figure 2A and Supplementary Figure 4) and nuclear DNA (data not shown) phylogenies was identified for AG1-IC lineage. In the phylogenetic tree of nuclear genomes, AG1-IC was most closely related to AG1-IB, and they formed a clade parallel with AG1-IA. In our previous RNA-seq analysis, we found that among AG1 strains, AG1-IB and AG1-IC had the most and least frequent polymorphisms, respectively (Yamamoto et al., 2019), which was consistent with our mitogenomic analysis, i.e., the comparison of sequences between AG1-IC and other strains with high substitution rates (Figure 3).

Positive selection signals in fungal mitochondrial *rps3* genes have been reported previously (Lin et al., 2015, 2017; Kang et al., 2017; Wang et al., 2020; Zhang et al., 2020; Huang et al., 2021; Wu et al., 2021). Together with *rps3*, genes encoding ribosomal subunits with positive AT and GC skewness are identified in the mitogenomes of brown rot fungal pathogens (Yildiz and Ozkilinc, 2021). In our results, we detected several sites in *R. solani rps3* genes representing positively selected signals. These sites may be the hot spot region in the *R. solani* mitogenomes and they may contribute to host adaptation.

Mitochondrial DNA has been popularly used to design markers for study of genetic diversity (Galtier et al., 2009), such as the study in medicinal fungus *Cordyceps militaris* (Zhang et al., 2017). However, to our knowledge, the used of DNA markers to investigate intraspecific genetic diversity of *Rhizoctonia* sp. are mainly designed from nuclear genomes (Das et al., 2020). With the increase in publication of *Rhizoctonia* mitogenomes from different AGs, the design of mitochondrial DNA markers for identification of pathogens will become possible. Meanwhile, our mitochondrial phylogeny including AG1-IA, AG1-IB, AG1-IC, AG3, AG4, and other *Rhizoctonia* strains that adapt to different hosts will acting as a phylogenetic marker to investigate host adaptation between AGs.

Additionally, the expression of mitogenome encoded genes may offer clues to understand host adaptation for *R. solani* strains in the future studies. Although the 15 typical protein-coding genes were highly conserved in the strains, their expression in AG1-IA and AG3 during rice and potato infection were quite different (Figures 5, 6 and Supplementary Figure 3). The AG1-IA has many plant hosts, including rice, corn, soybeans, barley, potatoes, and cabbage, while AG3 hosts are potatoes and tobacco (Yang and Li, 2012). The host infection process requires energy provided by mitochondria. To adapt to different host infection, gene regulation in mitochondria may be very complex. The different correlation coefficient maps in AG1-IA and AG3 showed the more complex relationships between genes in AG1-IA (Figure 6). Even for AG1-IA strains, the *atp8* gene from rice isolated strains was not expressed during rice infection, while the *atp8* gene from soybeans or corn isolated strains was expressed during rice infection (Supplementary Table 12). These gene repertoires may be difficult to explain currently, but the strain-specific phenomena of gene expression patterns were very interesting.

Gene expression is a fundamental life process, which is essential for fungal growth, metabolism, virulence, and response to environments. The comparison of expression patterns between RNA-seq and RT-qPCR analyses (Supplementary Figure 5) suggested the complex expression and regulation for genes, although similar patterns were found for several genes (such as *cob*, *rps3*, *mag28*, and *mag4*). Those highly expressed genes in rice infection (Figure 5), such as the *cox2* with an expression peak at 10-h, may play a significant role at the beginning of AG1-IA pathogenesis and may act as candidate targets for disease control. A comparison of amino acids between AG1-IA *cox2* and human *cox2* (i.e., *MT-CO2*) showed highly conserved sequences, with *E*-value of 2e-72 and identity of 46%. The sequence mutations in human *MT-CO2* have been reported to be related to serious diseases (Rahman et al., 1999; Heidari et al., 2020), suggesting that there may be also some potential key pathogenic factors in the *R. solani* mitogenome. The CRISPR gene-editing technology could facilitate genetic alterations in fungal genomes and enable study of gene function (Liu et al., 2015; Muñoz et al., 2019), in relation to changes in fungal growth, morphology, and virulence. Gene editing may also accelerate our understanding of the role of mitochondrial genes.

## Data Availability Statement

The datasets presented in this study can be found in the NCBI under the following accession numbers: MW995474.1 (https://www.ncbi.nlm.nih.gov/nuccore/MW995474.1/), MW995475.1 (https://www.ncbi.nlm.nih.gov/nuccore/MW995475.1/), and MW995476.1 (https://www.ncbi.nlm.nih.gov/nuccore/MW995476.1).

## Author Contributions

RL, YX, YL, and DZ contributed equally to this work. AZ designed the study. YX, DZ, and YL conducted the experiments. RL, YX, DZ, XX, XN, and AZ analyzed the mitogenomic data. RL, YX, DZ, YL, LJ, and XW analyzed the transcriptomic data. RL and AZ submitted the data to NCBI. RL, YX, YL, and AZ wrote the manuscript. All authors contributed to the article and approved the submitted version.

## Conflict of Interest

The authors declare that the research was conducted in the absence of any commercial or financial relationships that could be construed as a potential conflict of interest.

## Publisher’s Note

All claims expressed in this article are solely those of the authors and do not necessarily represent those of their affiliated organizations, or those of the publisher, the editors and the reviewers. Any product that may be evaluated in this article, or claim that may be made by its manufacturer, is not guaranteed or endorsed by the publisher.

## Footnotes

1 https://www.ncbi.nlm.nih.gov/genome/browse#!/organelles/; April 2021

2
http://www.repeatmasker.org/
