# Supplementary material for: Comparative Mitogenomic Analysis and the Evolution of Rhizoctonia solani Anastomosis Groups
Source: Front Microbiol. 2021 Sep 20;12:707281. doi: 10.3389/fmicb.2021.707281 (PMC8488467; doi:10.3389/fmicb.2021.707281)
Supplement: Supplementary file 1 [file Data_Sheet_1.PDF]

Supplementary Figures

AG4 *rps3* (JADHEA010000014.1:137644-138521:-)

```
ATGTCTTATA-TTTTTCTTCCTTAGGGGCTACGGAACATGCTAATAGAAAAAAAGCAAATAAAATTATCCAAAATTTTTACCTTAGA
M  S  Y  *  F  S  S  L  G  A  T  E  H  A  N  R  K  K  A  N  K  I  I  Q  N  F  Y  L  R
TTGTCTTCTCTAATTAGCACACCCTTTTATGCTATTACGCCTAGAACTGTTATAATAAGCTTATGTTTTTACTTTACCGAACAAAAT
L  S  S  L  I  S  T  P  F  Y  A  I  T  P  R  T  V  I  I  S  L  C  F  Y  F  T  E  Q  N
CTTAAATCATACTATGCTCTTACAGTGCCGCACCATAGTGCGTCTGATTTATCGACTTTAGCCGACACTAAAGATAAAATAAGGCTA
L  K  S  Y  Y  A  L  T  V  P  H  H  S  A  S  D  L  S  T  L  A  D  T  K  D  K  I  R  L
CAGCCGATCAACGAACCAAAAGGACCTCATGGTTTGGCACGGTTAGAGGACCCAGATTACTCACCTATTCTTGGGGCAATTCCGACT
Q  P  I  N  E  P  K  G  P  H  G  L  A  R  L  E  D  P  D  Y  S  P  I  L  G  A  I  P  T
GAAAAGTCCAGATTTAAACCTAATTTAGCGACAACGGTTAAGAATCACCACAGTACAAGCTTATTTAATTTAGGAACTGACAAATTT
E  K  S  R  F  K  P  N  L  A  T  T  V  K  N  H  H  S  T  S  L  F  N  L  G  T  D  K  F
CTTTTTAACTCTATTAGAACTCAAAAATTATTAGTTGTTTTAAGTAAATTATTCGGTAAAAATGTTAAATTGCAATTAGTGAGATTA
L  F  N  S  I  R  T  Q  K  L  L  V  V  L  S  K  L  F  G  K  N  V  K  L  Q  L  V  R  L
TACAACCCTTTTAAAGATAGTAAAATCTTAGCTCAACTTATTGGATTAAATGGTAAAGATTACAAATTCTATAAAATAGAAGATATG
Y  N  P  F  K  D  S  K  I  L  A  Q  L  I  G  L  N  G  K  D  Y  K  F  Y  K  I  E  D  M
CTATTTTCACGAGCAGTTATAAATAAAAAATTAAATAGAAATAGTAATTTAATCGAATTTACTCAAATAGCTTCTATCCTTAACGGA
L  F  S  R  A  V  I  N  K  K  L  N  R  N  S  N  L  I  E  F  T  Q  I  A  S  I  L  N  G
ATTAAAATCAGATTAGCGGGTAGACTTATAACTCAAAATATTGTACCTAAAAATACGGTAACTTTTGTTGAAAGAGGAGGATTCGCT
I  K  I  R  L  A  G  R  L  I  T  Q  N  I  V  P  K  N  T  V  T  F  V  E  R  G  G  F  A
AAAGGATATAACAACCTAAATGATTGCTCAACATTTACAACTAAAAATAAATTAGGGACTTATTCAGTTAAAGTATGGCTTTCCCAC
K  G  Y  N  N  L  N  D  C  S  T  F  T  T  K  N  K  L  G  T  Y  S  V  K  V  W  L  S  H
AAAGTAACTAACTAA
K  V  T  N
```

Supplementary Figure S1. The discovery of pseudogene *rps3* in the AG4 mitogenome. The mitogenome sequences of AG4 were shown in Supplementary Table S2.

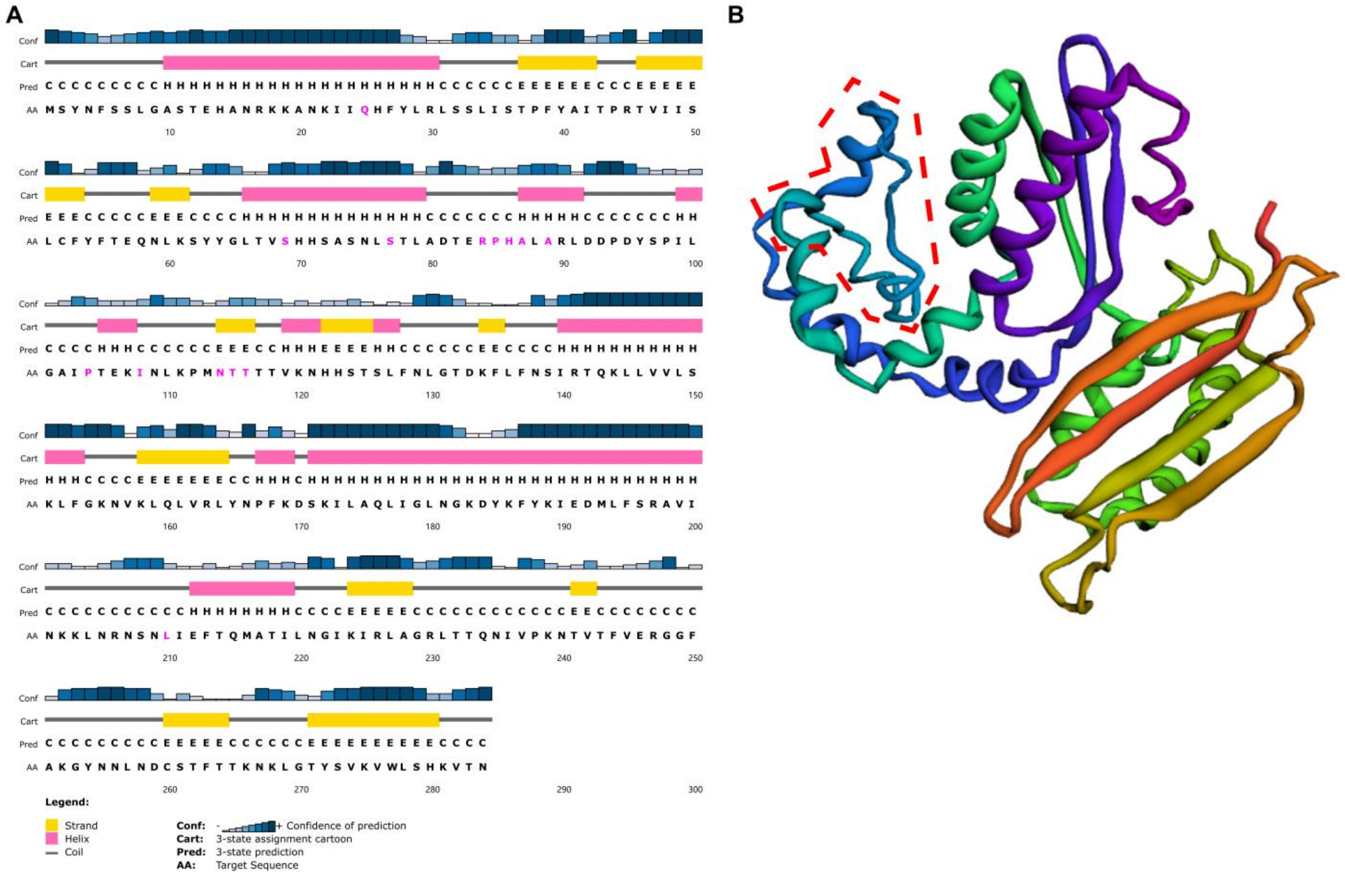

Supplementary Figure S2. The predicted protein structure of AG1-IA rps3. (A) The secondary structure map predicted by PSIPRED. The positively selected sites were marked in pink. (B) The protein structure predicted by RoseTTAFold with confidence of 0.46. The 84-116 aa with positively selected sites were indicated by the red dotted line circle.

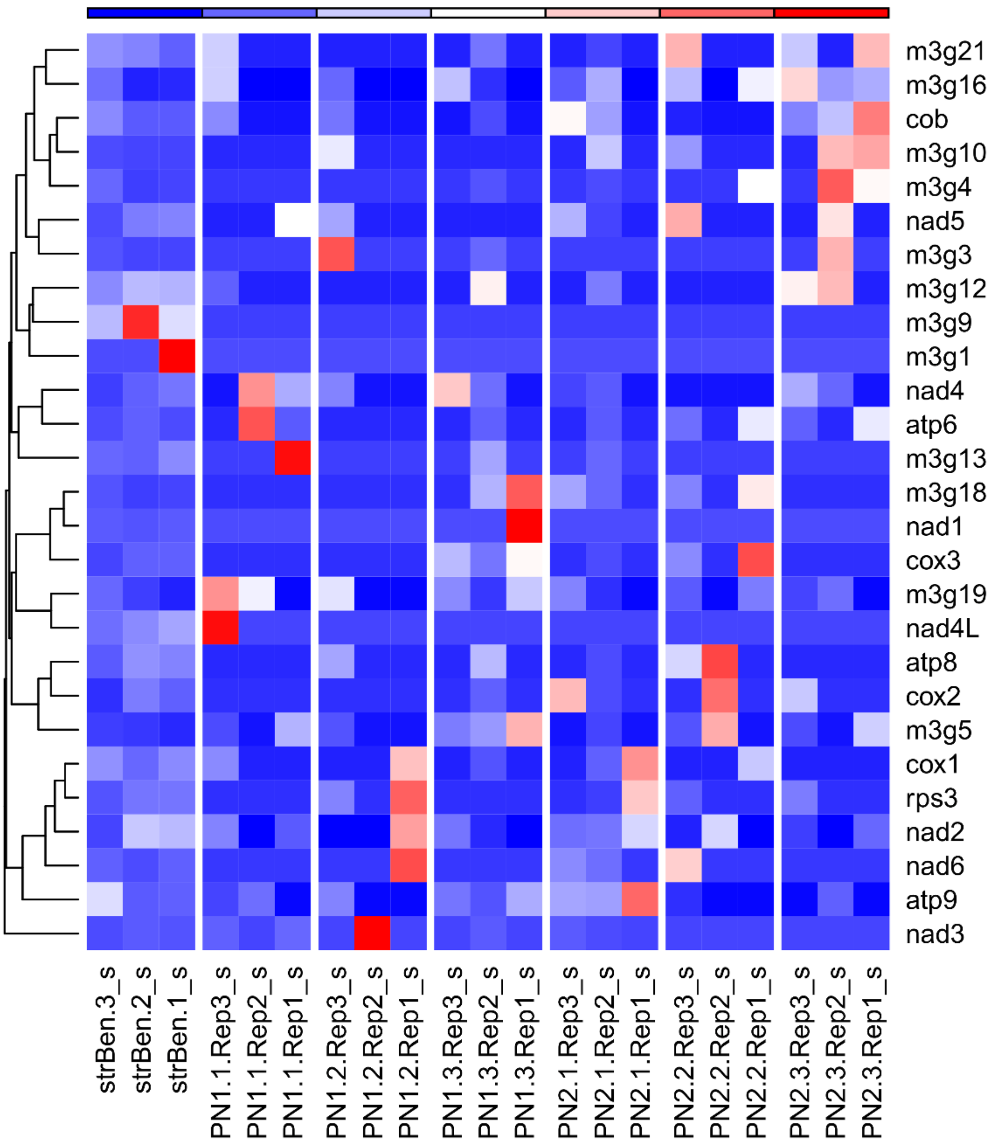

Supplementary Figure S3. Expression of genes from *R. solani* AG3 mitogenome.

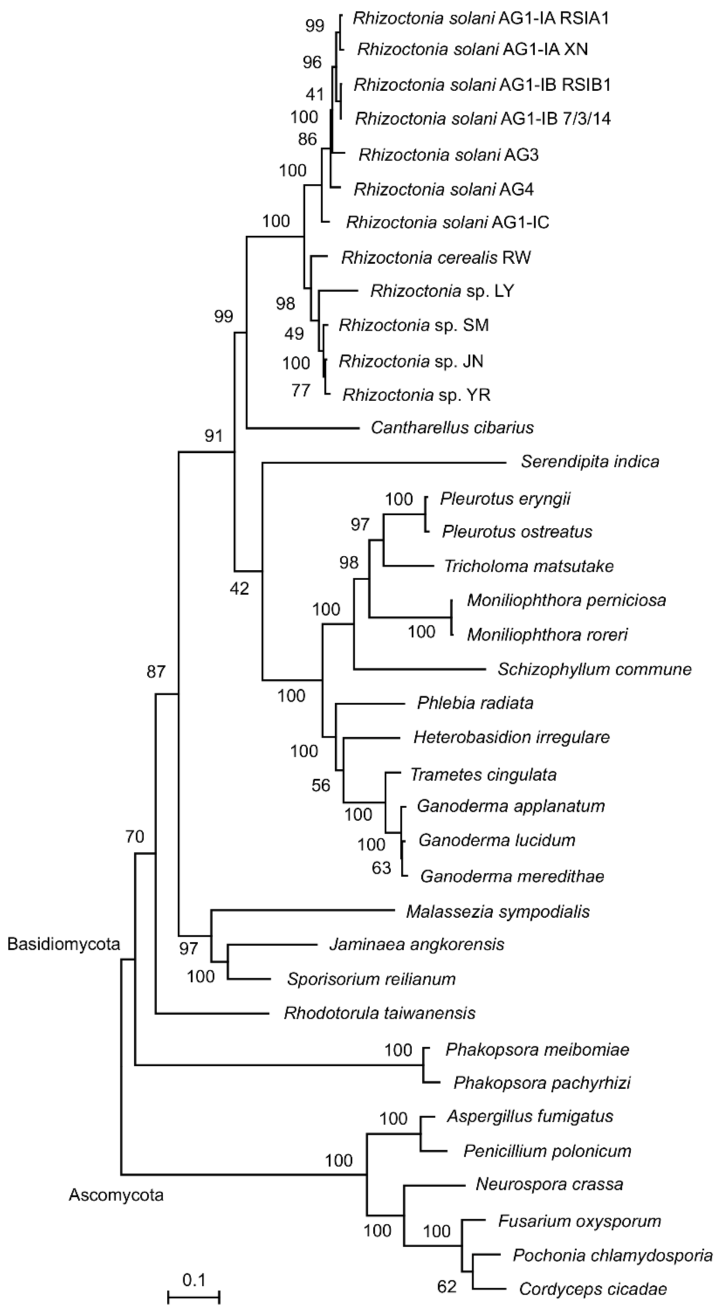

Supplementary Figure S4. The phylogenetic tree including mitogenomes from Basidiomycota and Ascomycota.

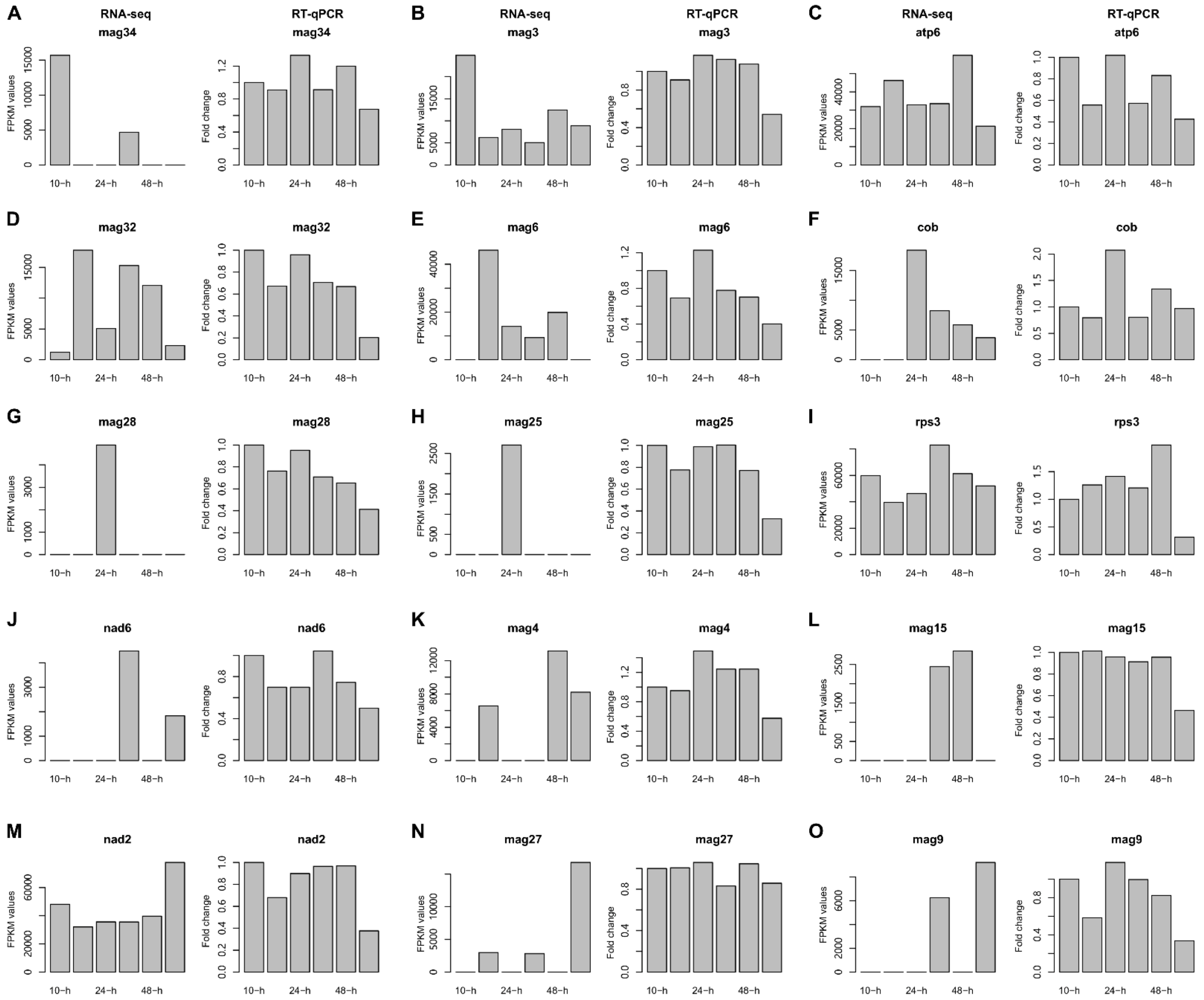

Supplementary Figure S5. Comparison of gene expression patterns between RNA-seq and RT-qPCR analyses. Six time points (10-h, 18-h, 24-h, 32-h, 48-h and 72-h) were included.
